# Response to fatigue observed through magnetic resonance imaging on the quadriceps muscle in postmenopausal women

**DOI:** 10.6061/clinics/2020/e1768

**Published:** 2020-06-24

**Authors:** Guilherme Carlos Brech, Thalita Sousa de Paula, Thiago Antônio Fedele, Aluane Silva Dias, José Maria Soares-Júnior, Marcelo Bordalo-Rodrigues, Edmund Chada Baracat, Angélica Castilho Alonso, Julia Maria D’Andréa Greve

**Affiliations:** I Laboratorio de Estudos do Movimento, Instituto de Ortopedia e Traumatologia (IOT), Hopital das Clinicas HCFMUSP, Faculdade de Medicina, Universidade de Sao Paulo, Sao Paulo, SP, BR; II Departamento de Fisioterapia, Universidade Nove de Julho (UNINOVE), Sao Paulo, SP, BR; III Programa de Ciencias do Envelhecimento, Universidade Sao Judas Tadeu (USJT), Sao Paulo, SP, BR; IV Radiologia, Instituto de Ortopedia e Traumatologia (IOT), Hospital das Clinicas HCFMUSP, Faculdade de Medicina, Universidade de Sao Paulo, Sao Paulo, SP, BR; V Disciplina de Ginecologia, Departamento de Obstetricia e Ginecologia, Hospital das Clinicas HCFMUSP, Faculdade de Medicina, Universidade de Sao Paulo, Sao Paulo, SP, BR

**Keywords:** Osteoporosis, Postmenopausal, Sarcopenia, Muscle Fatigue, Magnetic Resonance Imaging

## Abstract

**OBJECTIVES:**

Menopause marks the end of women’s reproductive period and can lead to sarcopenia and osteoporosis (OP), increasing the risk of falls and fractures. The aim of this study is to evaluate the influence of normal and low bone mineral density (BMD) on muscular activity, observed through inflammatory edema when mapping using magnetic resonance imaging (MRI) on the quadriceps muscle of postmenopausal women.

**METHODS:**

This was a cross-sectional study involving 16 older women, who were divided into two groups: osteoporosis group (OG), older women with OP, and control group (CG), older women without OP. The groups were evaluated in terms of nuclear MRI exam before and after carrying out fatigue protocol exercises using an isokinetic dynamometer and squatting exercises.

**RESULTS:**

The results of the present study showed that in intragroup comparisons, for both groups, there was a significant increase (*p*<0.05) in the T2 signal of the nuclear MRI in the quadriceps muscle after carrying out exercises using both thighs. In the intergroup comparison, no statistically significant difference was observed between the OG and CG, pre- (*p*=0.343) and postexercise (*p*=0.874).

**CONCLUSION:**

The acute muscular activation of the quadriceps evaluated by T2 mapping on nuclear MRI equipment is equal in women with and without OP in the postmenopausal phase. BMD did not interfere with muscle response to exercise when muscle fatigue was reached.

## INTRODUCTION

Menopause marks the end of the fertile phase of a woman’s life (1-3) and happens, on average, at 51 years of age in industrialized countries and 48 years of age in nonindustrialized countries (4). In the postmenopausal phase, defined as the period following 12 or more months of amenorrhea, begins the state of hypoestrogenism, which could result in the reduction of bone mass (2,5,6) and lead to osteoporosis (OP). OP is the reduction of bone mineral density (BMD), with changes in the microarchitecture of bones and an increased risk of fractures and/or bone deformities (2,4,7-10).

In addition to OP, another frequent manifestation during this period is vitamin D deficiency, expressed levels of plasma 25-hydroxyvitamin D [25(OH)D] levels, due to its role in controlling various hormones that act on bones and muscles, modulating their interrelationships (11-13). The vitamin deficiency results in the reduction of BMD and sarcopenia, which is characterized by the reduction of muscular mass and strength (hypotrophy of type 2 fibers - fast twitch and strength) with the resulting deficit in physical performance and increased risk of falls (9,10,14-20).

Nuclear magnetic resonance imaging (MRI) is a diagnostic imaging tool that has proved to be an effective method for muscular analysis by measuring the quantitative values of the metabolic dynamic of skeletal muscles during degenerative conditions, inflammatory responses, regenerative processes, and after exercise (21,22). MRI can be weighted in T2 (transversal relaxation time) and T1 (longitudinal relaxation time), and the nature of the image contrast is based on relative contributions from different tissues. Alterations in the T2 signal, seen on the “T2 map,” are caused by the acute increase in muscular activity and its relation to the relaxation time of T2, which depends on the presence of water in the muscle. The signal intensification is caused by the osmotic motility of intramuscular water protons that enter the cells because of the increase in acidosis and in the intracellular space, caused by muscular catabolites (21-24). MRI measures the movement of intramuscular water and its repercussion on muscular metabolism, which can be used as parameters when evaluating muscular activation.

It is known that the quadriceps muscle plays a fundamental role in maintaining postural balance and in daily life activities: getting up from a chair, walking, climbing a staircase, and others. However, with advancing age and the presence of sarcopenia, there is a greater risk of falls and fractures, especially in postmenopausal women with OP (25). Furthermore, it is not known if the behavior of the quadriceps after exercise, when it is fatigued, would be worse in this population, a factor that would further aggravate the risk of falls. Therefore, it is important to clearly understand the muscular behavior of this population.

The main objective of this study was to evaluate the influence of BMD on muscular activity, observed through inflammatory edema via MRI mapping of the quadriceps muscle in postmenopausal women. A secondary objective was to analyze the correlation between 25(OH)D and muscular activity, observed through inflammatory edema via MRI mapping of the quadriceps muscle in postmenopausal women.

## METHODS

### Study location and ethical issues

The study was developed at the Kinesiology Laboratory and the Radiologic Division of the Institute of Orthopedics and Traumatology, together with the Gynecology and Obstetrics Department at the Clinic Hospital of the University of São Paulo Medical School (HCFMUSP), with approval from the Ethics Committee of the University of São Paulo (registration number 10785/2013).

### Type of study and subjects

This was a cross-sectional study involving 16 elderly women, which were divided into two groups: osteoporosis group (OG), elderly women with OP, and control group (CG), elderly women without OP. The inclusion criteria were as follows: postmenopausal period with 12 or more months of amenorrhea; aged 55 to 65 years; classified as active, irregularly active, or sedentary according to the International Physical Activity Questionnaire (IPAQ) results; has not done regular physical activity for at least the previous 3 months; absence of lesions, diseases, or trauma to the lower limbs in the previous 6 months; does not present any limitations of joint movement and/or deformity of the knee and hip; can walk independently without aid and without claudication for at least 100 meters; is independent in their activities of daily living; does not use a pacemaker; has metallic implants or suffers from claustrophobia, all of which impede the use of the MRI; does not take medication such as thyroid hormones, estrogen, or herbal substances, antidepressants, and diuretics; and absence of endocrine diseases such as hyperparathyroidism, diabetes, hypertension, hypothyroidism, and hyperprolactinemia. The OG had an OP diagnosis according to BMD (T-score≥-2.5). The CG does not present with a diagnosis of OP and osteopenia according to BMD (T-score≤-1.0). The exclusion criteria were as follows: inability to carry out one of the tests, systolic pressure equal to or higher than 160mmHg and diastolic pressure equal to or higher than 120mmHG at the time of testing, and mention of pain during the performance of the isokinetic test.

The volunteers in the OG were selected from the Gynecological, Endocrinological, and Climacteric outpatient facility at the Central Institute of the Clinic Hospital of the Medical College of the University of São Paulo, and the control women were recruited from elders in community groups.

The demographic characteristics of the groups are presented in Table 1.

### Procedures

The groups were evaluated in terms of BMD, blood test for 25(OH)D levels, nuclear magnetic resonance exam, and fatigue protocol on isokinetic dynamometer and squatting exercise.

#### Bone densitometry

All volunteers submitted to bone densitometry during evaluation, if they had not had a recent exam. Exams were accepted if they were carried out within the last 12 months or more recently, to determine the group into which they would be included based on BMD (8,16,19).

#### 25(OH)D

One milliliter of blood was collected from each subject and stored in a test tube to measure the serum 25(OH)D and calcium levels. The LIAISON 25 OH Vitamin D Total Assay kit (DiaSorin Inc., MN, USA) was used with the direct competitive chemiluminescent immunoassay for the quantitative determination of the total serum 25(OH)D levels, which were used to evaluate the occurrence of muscular fatigue after the exercises.

#### Nuclear magnetic resonance exam

The volunteers underwent two MRI exams of the right and left thighs, at two distinct moments: before and after the fatigue protocol exercises of the quadriceps muscle, that is, the preexercise MRI, with prior rest of 30 or more minutes to determine the base values for comparing T2 mapping before the fatigue protocol exercises of the quadriceps muscle, and the postexercise MRI, carried out immediately after the fatigue protocol exercises of the quadriceps muscle. Inflammatory edema, induced by the fatigue protocol exercises of the quadriceps muscle, was evaluated via T2 mapping, in a comparison with the first exam. To carry out the MRI examination, volunteers were in supine position on a sliding table attached to the MRI machine (1.5T MRI system from General Electric Company (GE), model HDxt) with the bilateral coxofemoral region positioned inside the 8 channels Body Full-HD coil.

The following parameters were used for pre- and post-fatigue protocol evaluations: T2 map (Fiel of view (FOB) 38, thickness/spacing of cuts 8.0mm/1.0mm, matrix 256×256, Number of excitations (NEX) 1.0, TR 1750, multi-TE 8.0, BDW 41, 24 cuts); axial Short-TI Inversion Recovery (FOV 38, thickness/spacing of cuts 8.0mm/1.0mm, matrix 320×256, NEX 2.0, TR 4525, TE17, BW 31), axial T1 (FOV 38, thickness/spacing of cuts 8.0mm/1.0mm, matrix 352×256, NEX 2.0, TR 767, TE minimum, BW 41, 24 cuts), and coronal T1 (FOV 48, thickness/spacing of cuts 5.0mm/1.0mm, matrix 384×256, NEX 1.0, TR 667, TE minimum, BDW 62, 24 cuts).

**Figure 1:**
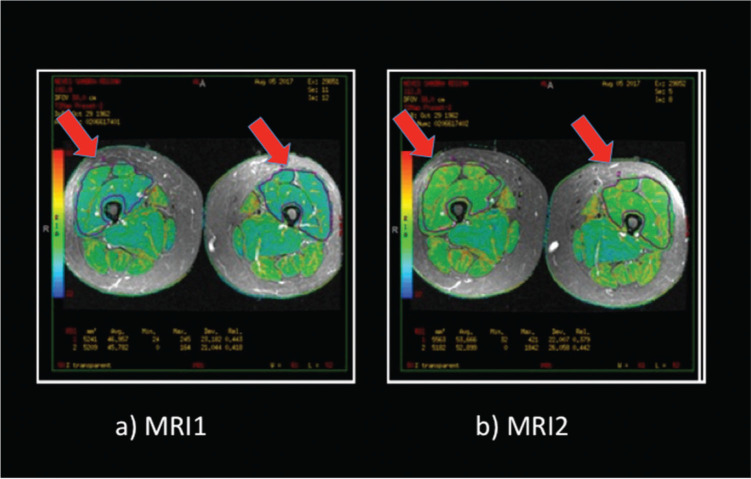
Nuclear magnetic resonance imaging (a) in preexercise period (MRI1) and (b) in postexercise period (fatigue) of the right and left thighs (MRI2). Indicated area corresponds to the quadriceps muscle.

Image processing was carried out for each sequence of T2 mapping on an Advantage Workstation for Diagnostic Imaging Post processing 4.2 GE computer. The (bilateral) region of interest of the muscular region of the quadriceps (ROI) was selected, and the ROIs were drawn, excluding the skin, subcutaneous tissue, bone, blood vessels, and fat via the Short-TI Inversion Recovery sequence (17,26) (Figure 1).

#### Femoral quadriceps muscle fatigue protocol

With the objective of causing fatigue in the femoral quadriceps muscle, two series of 10 repetitions were carried out, with a 1-minute interval between each series, with the angular velocity of 180° of flexion and extension of the knee on the isokinetic dynamometer using the Biodex^®^ Multi-Joint System 3 (Biodex Medical^TM^, Shirley, NY, USA). The isokinetic dynamometer was calibrated 30 minutes before starting the tests. After a standardized warm-up, the women were positioned for concentric evaluation of extension and flexion movements of the knee joint. They remained seated with the hips at 90° of flexion and secured to the chair by belts. The test was started with the dominant limb. The limb was evaluated by positioning the lateral condyle of the femur in alignment with the mechanical axis of the dynamometer. All women performed four submaximal repetitions to become familiar with the equipment, followed by a 60-s rest interval and then two series of five maximal repetitions of knee extension and flexion starting with the dominant limb, with a 60-s interval between the series. Constant standardized verbal encouragement was given during the tests to promote maximum effort during contractions (27,28). Heart rate and blood pressure were controlled before and after the tests. This protocol was based on literature recommending exercise with angular speed and a larger number of repetitions, with the aim of causing muscle fatigue (29,30).

In addition to the isokinetic dynamometer, to ensure the maintenance of muscular fatigue until the postexercise MRI could be carried out, volunteers were asked to step up and down from a 10-cm-high step for five minutes and carry out three squats, maintaining the posterior part of the trunk supported against the wall, and, after the last squat, carry out an isometric contraction of the quadriceps for 1 min or until they noted fatigue. Blood samples were collected for lactate dosing using the Lactate Roche Accutrend^®^ Lactimeter, during rest (Lac1), 1 min after the exercises (Lac2), and 3 min after the exercises (Lac3) to establish the presence of muscular fatigue, and following that, patients were submitted to the second MRI exam.

#### Statistical analysis

The data were stored and analyzed on the IBM SPSS Statistics 22 program. A descriptive analysis of the data was carried out using frequency tables for the qualitative variables and measures of position and dispersion for the quantitative variables. The normality and homogeneity of data variance were confirmed using the Shapiro-Wilk test. The data were reported by calculating the average and standard deviation.

The variance test (ANOVA two-way) was used as means between groups, being the factors moment (pre- *vs* post-intervention) and group (OG *vs* CG). The effect size was calculated by computing the eta partial square (η2). The results from the effect size were analyzed according to the following criteria: small (d=0.02), medium (d=0.13), and large (d=0.26) (31).

For correlations between the postexercise MRI results and 25(OH)D levels, the Pearson correlation test was used.

The significance level was set at *p*<0.05.

## RESULTS

The results of this study show that, in the comparison between Lac1 (resting lactate levels) and Lac3 (lactate levels 3 min after executing the exercises), it was noted that both groups, OG (*p*<0.0001) and CG (*p*<0.0001), reached fatigue through the isokinetic dynamometer exercises and in the squatting exercises.

Table 2 presents the T2 map of MRI data in the pre- and postexercise periods for groups in both quadriceps sides. There was a significant increase in the T2 map of MRI data in the right quadriceps (F_1,31_=10.578; *p*=0.03; η=0.27) and left quadriceps (F_1,31_=17.042; *p*≤0.001; η=0.37) before and after the fatigue protocol. There were no differences between groups in any of the quadriceps side, the right (F_1,31_=0.649; *p*=0.47; η=0.02) and left sides (F_1,31_=0.095; *p*=0.76; η=0.03). Also, no differences were observed between the moment in any of the quadriceps side, the right (F_1,31_=0.018; *p*=0.89; η=0.1) and left sides (F_1,31_=0.096; *p*=0.75; η=0.03).

**Figure 2:**
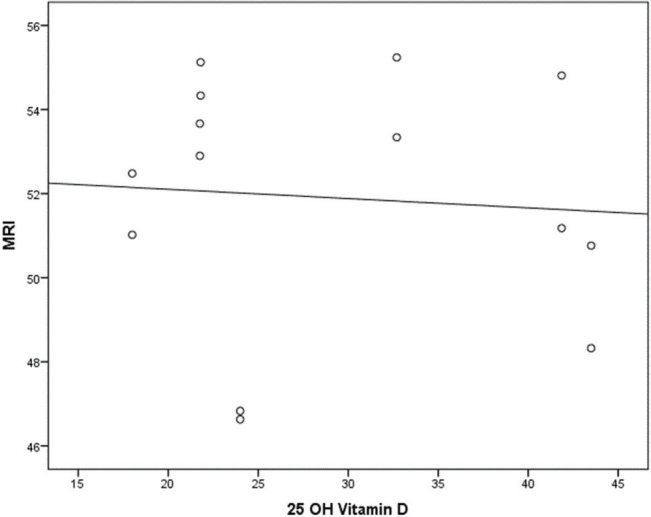
Correlation between average bilateral MRI (postexercise/fatigue) and 25-OH vitamin D levels.

The results of the 25(OH)D levels (Table 1) were correlated to the average measure for both legs obtained in MRI T2 (Figure 2). There was no correlation between the 25(OH)D level and muscular activity observed through inflammatory edema in MRI mapping (r=-0.171; *p*=0.525).

## DISCUSSION

This study found that in a comparison of MRI results between the thighs of both groups studied, there was no significant difference before and after exercises.

The T2 activation signal increased in both groups, but there was no significant difference between the two groups.

No studies were found in the literature that evaluated muscular metabolic changes via the T2 map in women with and without OP in the postmenopausal period using MR images to serve as a base of comparison for this study.

The exercise protocol used caused fatigue in the knee extensor muscles, as there was an increase in lactic acid levels in both measures taken after carrying out the exercises. Lactate is generated as a result of anaerobic glycolysis by all body tissues, serving as a marker of the index of fatigue following exercise (32,33). Baker et al. (33) investigated blood lactate levels after 10 and 20 s of maximum activity on the cycle ergometer and noted a difference, with an increase in the dose of lactate, using the same method used in this study.

Mendiguchia et al. (34) studied posterior thigh muscles of soccer players and showed that MRIs are an effective method to capture the acute post-practice muscular changes through the T2 signal. Meanwhile, Mendiguchia et al. (34) showed greater metabolic activity in recto-femoral muscles, vastus medialis, and gluteus maximus using the MRI T2 map immediately after training using the Pilates Method. Both studies are similar to the present study, which showed an increase in the T2 map after a series of exercises of the quadriceps muscle, a finding that indicates an increase in metabolic activity of muscle cells. Therefore, we can consider the T2 map to serve as a sensible tool for evaluating acute metabolic changes of muscles following a session of exercises, leading to muscle fatigue.

Regarding the level of physical activity, all participants were classified as active or irregularly active, according to the IPAQ (35). However, none of them practiced any physical activity or sport regularly, a factor that led to homogeneity of the samples. Azzabou et al. (17) showed that age is an important factor in MRI muscle changes, but did not find any correlation with levels of physical activity.

Age and OP create a predisposition to important morphological changes such as sarcopenia. Aging is directly related to the progressive reduction of muscle mass, and there is an association between sarcopenia and OP, which increases in postmenopausal women as a result of the accompanying hormonal changes (36).

MRIs could be considered an important, noninvasive tool for muscular evaluations (24). It is an instrument capable of capturing changes in metabolic activity of muscle cells through the increased intensity of the T2 signal (17).

However, the results of this study showed that there was no difference in the muscular activation of women with and without OP, indicating that there was no necessary association between bone mass loss and sarcopenia, at least in women with characteristics similar to the sample studied. These findings differ from data in the literature, which shows a strong correlation between muscle and bone changes (36) for this same studied population. Therefore, the results contradict the initial hypothesis of the study, but are promising in terms of indicating exercises for women with OP, because they can expect good muscle response to those exercises. This is a factor that could improve the evolution of sarcopenia and OP, as well as help prevent frailty and its consequences, such as falls and fractures, factors that play an important role in postmenopausal women’s health (18).

As for the 25(OH)D deficiency, we know that it can be directly related to the hypotrophy of type 2 fibers (14,) through its modulating action in the control of hormones that affect bones and muscles (12,13). For this reason, although the sample size is small, a correlation was found between 25(OH)D levels and the T2 map, but significant data were not found. The absence of correlations between these variables may indicate that 25(OH)D levels in the sample studied were not very low. For this reason, care was taken to ensure that all participants, as part of the inclusion criteria, were not ingesting 25(OH)D supplements at the time of evaluation because supplements could interfere in the results (10,16,18,36).

This study has important clinical implications, showing that MRIs can be a precise instrument in evaluating inflammatory edema through the mapping of muscular structures.

In the age group studied, even in women with OP, changes that resulted from sarcopenia had not yet set in and did not influence muscular metabolism. However, it is necessary to carry out studies that do not present the same limitations as the present study in terms of sample size and the absence of bone densitometry for body composition, which could help in the understanding and quantification of lean tissue.

## CONCLUSION

The acute muscular activation of the quadriceps evaluated using T2 mapping on an MRI machine is similar in women with and without OP in the postmenopausal phase. BMD did not interfere in muscle response to exercise after reaching muscle fatigue.

## AUTHOR CONTRIBUTIONS

Brech GC and Paula TS were responsible for the investigation and manuscript original drafting. Fedele TA, Dias AS and Bordalo-Rodrigues M were responsible for the investigation. Soares-Júnior JM and Baracat EC were responsible for the formal analysis and manuscript review. Alonso AC was responsible for the manuscript writing, editing and review. Greve JMA supervised the study and was responsible for the manuscript writing, editing and review.

## Figures and Tables

**Table 1:** Baseline demographic characteristics.

	OG	CG	*p*
N	9	7	
Age (years)	61.33 (±3.57)	61.86 (±2.61)	*p*=0.746
Time of menopause (years)	13.2 (±3.3)	13.00 (±4.83)	
Body mass (Kg)	60.26 (±11.89)	69.40 (±16.13)	*p*=0.212
Height (m)	1.52 (±0.04)	1.55 (±0.75)	*p*=0.337
BMI (kg/m^2^)	25.90 (±4.15)	28.34 (±3.45)	*p*=0.232
Bone densitometry			
L1-L4			
% T-score	-3.01 (±0.41)	0.11 (±0.66)	* ***p*** **<0.001**
Femoral neck			
% T-score	-1.81 (±0.67)	1.10 (±1.97)	* ***p*** **=0.001**
Total femur			
% T-score	-1.27 (±0.56)	0.61 (±0.38)	* ***p*** **<0.001**
25-OH vitamin D			
Level (ng/mL)	29.50±(5.83)	29.08±(10.32)	*p*=0.927
Lactate level (moments)			
Lac1	2.90 (±0.59)	2.38±(0.71)	*p*=0.742
Lac2	5.52±(1.16)	3.87±(1.07)	***p*** **=0.018**
Lac3	6.31±0(.76)	4.35±(1.15)	***p*** **=0.002**

OG = osteoporosis group, CG=control group, N=sample (number of individuals), BMI=body mass index, Lac1=at rest, Lac2=1-min after exercises, Lac3=3 minutes after exercises, T-student. *p≤0.05.

**Table 2:** T2 map of MRI in pre- and postexercise periods for groups in both quadriceps sides.

	Osteoporosis group		Control group	
	Preexercise M (sd)	Postexercise M (sd)	Preexercise M (sd)	Postexercise M (sd)
**Right**	47.1 (3.0)	51.1 (3.8)^a^	48.0 (4.3)	52.3 (3.0)^a^
**Left**	46.8 (2.9)	51.4 (3.7)^a^	46.0 (3.8)	51.4 (2.9)^a^

T2=relaxation time signal, MRI=magnetic resonance imaging, M=mean, SD=standard deviation. ANOVA two-way a ≠ pre *versus* post for *p*≤0.05.
